# High *Plasmodium* Infection Rate and Reduced Bed Net Efficacy in Multiple Insecticide-Resistant Malaria Vectors in Kinshasa, Democratic Republic of Congo

**DOI:** 10.1093/infdis/jix570

**Published:** 2017-10-26

**Authors:** Jacob M Riveron, Francis Watsenga, Helen Irving, Seth R Irish, Charles S Wondji

**Affiliations:** 1 Vector Biology Department, Liverpool School of Tropical Medicine, United Kingdom; 2 Research Unit, Liverpool School of Tropical Medicine (LSTM)/Organisation de Coordination pour la lutte contre les Endémies en Afrique Centrale (OCEAC) Research Unit, Yaoundé, Cameroon; 3 Institut National de Recherche Biomédicale, Kinshasa, Democratic Republic of Congo; 4 US President’s Malaria Initiative, Entomology Branch, Division of Parasitic Diseases and Malaria, Centers for Disease Control and Prevention, Atlanta, Georgia

**Keywords:** malaria, *Plasmodium falciparum*, insecticide resistance, Anopheles, Democratic Republic of Congo

## Abstract

Accounting for approximately 11% of all malaria cases, the Democratic Republic of the Congo (DRC) is central to malaria elimination efforts. To support vector control interventions in DRC, we characterized the dynamics and impact of insecticide resistance in major malaria vectors in 2015. High *Plasmodium* infection rates were recorded in *Anopheles gambiae* and *Anopheles funestus*, with *Plasmodium falciparum* predominant over *Plasmodium malariae*. Both mosquito species exhibited high and multiple resistance to major public health insecticide classes. The extremely high resistance to permethrin and DDT (dichlorodiphenyltrichloroethane) in *An. gambiae* (low mortalities after 6 hours exposure) is worrisome, and is supported by a reduced insecticidal effect of bed nets against both mosquito species in laboratory tests. Metabolic and target site insensitivity mechanisms are driving this resistance in *An. gambiae*, but only the former was observed in *An. funestus*. These findings highlight the urgent need for actions to prolong the effectiveness of insecticide-based interventions in DRC.

## Teaser

High and multiple resistance to insecticides are recorded in the 2 main malaria vectors in the Democratic Republic of the Congo, leading to a significant loss of efficacy of conventional bed nets in the presence of alarmingly high *Plasmodium* infection rate, suggesting high malaria transmission.

With 11% of all worldwide malaria cases occurring in the Democratic Republic of the Congo (DRC) [1], this country is key for malaria elimination. Control efforts in DRC extensively rely on the use of long-lasting insecticidal nets (LLINs). However, the development of insecticide resistance is threatening the effectiveness of this control tool, calling for urgent action to implement suitable resistance management strategies [2]. Unfortunately, the scarcity of information on the extent and impact of resistance prevents the design of such strategies in DRC.

Resistance to pyrethroids and organochlorines has previously been reported in DRC, although exclusively in the major malaria vector *Anopheles gambiae* [1, 3, 4]. Nevertheless, the extent and intensity of this resistance remain unclear and completely unknown for other major vectors such as *Anopheles funestus.* Additionally, the resistance profile to other insecticide classes has not been clearly established, limiting the ability of national malaria control programs to make informed decisions for resistance management. Indeed, so far, only limited evidence of resistance to carbamates and organophosphates has been reported in *An. gambiae* from DRC [1], with the exception of possible resistance to malathion observed in a single locality [3]. Furthermore, it also remains unclear how pyrethroid resistance in malaria vectors currently impacts the efficacy of both conventional and piperonyl butoxide (PBO)–based LLINs, although a low efficacy has been reported previously for the OlysetNet [5].

Limited investigation of resistance mechanisms in DRC has detected the presence of the *kdr* mutation in *An. gambiae* in Kinshasa [5]. The role of metabolic resistance has not been explored further. To support and facilitate the success of the ongoing and future vector control programs in DRC, the present study extensively evaluated the current insecticide resistance profile of *An. gambiae* and *An. funestus*, the 2 main malaria vectors in Kinshasa, the capital city. Furthermore, the efficacy of several LLINs was assessed, in addition to the molecular mechanisms driving resistance. Additionally, the *Plasmodium* infection rate in malaria vectors was also assessed.

## METHODS

### Study Area and Mosquito Sampling

Indoor-resting mosquitoes were collected using electrical aspirators from 10–15 households each day for a week at Ndjili-Brasserie, a suburb of Kinshasa (4°19′39″S, 15°18′48″E), in May 2015. This collection site was chosen due to the presence of suitable breeding sites for the 2 main malaria vectors, *An. gambiae* and *An. funestus*, through the Ndjili River and its flooded shores. Blood-fed and half-gravid female *Anopheles*, morphologically identified as belonging to the *An. funestus* or *An. gambiae* complex [6], were kept in holding cages for 4–6 days and forced to lay eggs individually during 2–3 extra days [7]. One hundred thirty-three *An. gambiae* sensu lato (s.l.) and 79 *An. funestus* s.l. laid eggs. F_0_ dead females and F_1_ eggs were transported to the Liverpool School of Tropical Medicine for the subsequent analysis under a license from the Department for Environment, Food and Rural Affairs (DEFRA) (PATH/125/2012, UK).

### Species Identification

Genomic DNA from 111 *An. gambiae* s.l. and 81 F_0_*An. funestus* s.l. whole female mosquitoes was extracted, using the Livak protocol [8], and identified to species level using a cocktail polymerase chain reaction (PCR) assay [9, 10]. Larvae were transferred to plastic trays according to species group for rearing, as previously described [7, 11].

### Plasmodium Infection Rate

The *Plasmodium* infection rate was estimated using the mosquito’s whole body extracts by detecting the presence of *Plasmodium falciparum* (F+) and/or *Plasmodium ovale*, *Plasmodium vivax*, and *Plasmodium malariae* (OVM+) in 109 *An. gambiae* s.l. and 81 *An. funestus* sensu stricto (s.s.) field-collected F_0_ females individually using the TaqMan assay, as previously described [12, 13]. Results of TaqMan assay were confirmed by performing a nested PCR assay as previously described [14].

### Insecticide Susceptibility Assays

World Health Organization (WHO) tube bioassays were performed using the F_1_ generation to assess the insecticide resistance profile of *An. gambiae* s.l. and *An. funestus* s.s. following WHO guidelines [15]. Insecticides tested include permethrin (0.75%), deltamethrin (0.05%), bendiocarb (0.1%), dichlorodiphenyltrichloroethane (DDT) (4%), malathion (5%), and fenitrothion (1%). Due to a low number of *An. funestus* s.s. F_1_ obtained, only males were tested for malathion and fenitrothion. Additionally, *An. gambiae* s.l. mosquitoes were exposed to λ-cyhalothrin (0.05%), etofenprox (0.05%), propoxur (0.1%), dieldrin (4%), and pirimiphos-methyl (1%). Control mosquitoes were exposed to papers impregnated only with insecticide carrier oil. The mortality rates were determined 24 hours later. Efficacy of all insecticide-impregnated papers was confirmed using the *An. gambiae* Kisumu susceptible laboratory strain.

WHO tube bioassays were used to assess the mortality of *An. gambiae* s.l. after extended periods (3 and 6 hours) of exposure to DDT and deltamethrin. Furthermore, in addition to the 60-minute exposure described above, mortality after 30- and 90-minute exposures to bendiocarb was also assessed. The mortality rates were determined 24 hours later.

### Synergist Assays

Synergist assays were performed with PBO using *An. gambiae* s.l. Four replicates of 20–25 adult mosquitoes (2–5 days old) were preexposed using WHO tube bioassays with PBO-impregnated papers (4 %) for 1 hour. Thereafter, the mosquitoes were immediately exposed to permethrin (0.75%), deltamethrin (0.05%), or DDT (4%) for 60 minutes. Control assays using only 4% PBO papers for 60 minutes were also performed. Mortality was scored after 24 hours, and the results obtained were compared with the mortality without PBO exposure using unpaired Student *t* test.

### Insecticide-Treated Bed Net Efficacy

The efficacy of conventional bed nets against both mosquito populations was estimated by 3-minute exposure cone bioassays following the WHO guidelines with minor modifications, with respect to the number of pieces of net and the number of mosquito per replicate tested [16]. In brief, 5 replicates of 10 F_1_ females (2–5 days old) were placed in plastic cones attached to different commercially produced nets newly purchased: OlysetNet, OlysetPlus, Permanet 2.0, Permanet 3.0-side and -roof, and an untreated net (as a control). Due to the low number of F_1_ mosquitoes available, only 1 piece per type of net was tested. After exposure, the mosquitoes were placed in holding paper cups with cotton soaked in 10% sugar solution. Mortality was recorded 24 hours after exposure.

### Polymorphism Analysis of the Voltage-Gated Sodium Channel Gene in *An. gambiae*

To assess the role of target-site knockdown resistance in *An. gambiae* s.l., 111 F_0_ female field-collected mosquitoes were genotyped for the L1014F and L1014S *kdr* mutations by TaqMan assay as previously described [17]. Furthermore, the genetic diversity of the voltage-gated sodium channel (VGSC) gene was investigated for *An. gambiae*. A fragment of intron 19 of the VGSC gene spanning a portion of exon 20 (including the 1014 codon associated with *kdr*) was amplified in 11 field-collected *An. gambiae* s.s. females, cleaned, and sequenced as previously described [7, 18]. Sequences were aligned using ClustalW [19], whereas haplotype reconstruction and polymorphism analysis were done using DnaSPv5.10 [20]. DRC haplotypes were compared to the 4 *kdr* haplotypes previously detected across Africa as containing either the 1014F (H1/H4) or the 1014S (H2/H3) mutations [21], as well as to a susceptible haplotype from Cameroon [22]. All DNA sequences have been submitted to GenBank (accession numbers KY700707–KY700728).

### Genotyping of Resistance Markers in *An. funestus*

The role of the *An. funestus* s.s. L119F-GSTe2 and A296S-RDL resistant markers, involved in DDT/permethrin and dieldrin resistance, respectively, was also evaluated by TaqMan assay, genotyping 66 F_0_ female mosquitoes collected from the field. L119F-GSTe2 and A296S-RDL TaqMan reactions were performed as previously described [23, 24].

### Transcription Profile of Resistance Genes in *An. funestus*

Total RNA was extracted from 3 batches of 10 adults from F_1_ female *An. funestus* s.s. mosquitoes nonexposed to insecticides and the FANG susceptible strain, as previously described [24, 25]. The expression patterns of key resistance genes including *CYP6P9a*, *CYP6P9b*, *CYP6M7*, and *GSTe2* were assessed by quantitative reverse-transcription PCR (qRT-PCR) [24]. After normalization with housekeeping genes *Actin* (AFUN006819) and *RSP7* (AFUN007153-RA), the relative expression for each gene was calculated according to the 2^-ΔΔCT^ method [26]. The statistical significance between gene expression estimates was performed using unpaired Student *t* test.

## RESULTS

All *An. funestus* group F_0_ mosquitoes (81) were confirmed to belong to *An. funestus* s.s., whereas the majority of a subsample of 100 F_0_*An. gambiae* s.l. tested were *An. gambiae* s.s. (95 of 100), with a low percentage of *Anopheles coluzzii* (5 of 100 [5%]).

### Plasmodium Infection Rate

Using TaqMan assays, 38 *An. gambiae* s.l. mosquitoes were found to be infected only with *P. falciparum* (F+; 34.9% [38/109]), whereas 3 mosquitoes were infected with *P. ovale*, *P. vivax*, and/or *P. malariae* (OVM+; 2.8% [3/109]); 4 mosquitoes were coinfected (F+/OVM+; 3.7% [4/109]). The nested PCR confirmed 31 F+ positive mosquitoes and that 2 F+/OVM+ mosquitoes were coinfected with *P. falciparum* and *P. malariae*. However, the nested PCR failed to confirm 8 F+ positive mosquitoes, the 3 OVM+ and 2 F+/OVM+ TaqMan-positive mosquitoes, probably because of the lower sensitivity of this method [12].

In the case of *An. funestus* s.s., 18 mosquitoes were infected only with *P. falciparum* (22.2% [18/81]), 3 mosquitoes were infected with OVM+ (3.7% [3/81]), and 4 mosquitoes were coinfected: F+/OVM+ (4.9% [4/81]). The nested PCR confirmed all *P. falciparum*–positive infections of *An. funestus* s.s. and established that the 3 OVM+ and 3 F+/OVM+ mosquitoes were infected/coinfected with *P. malariae*, while only 1 F+/OVM+ mosquito was coinfected with *P. ovale*.

### Insecticide Susceptibility Assays

**Figure 1.**
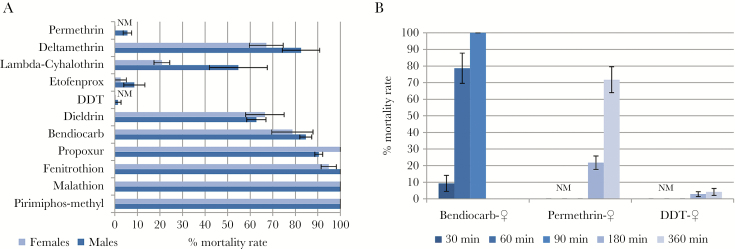
Susceptibility profile of *Anopheles gambiae* sensu lato population in Kinshasa using World Health Organization insecticide susceptibility tube assays with 60 minutes of exposure (*A*) and for females at different time points (*B*). Error bars represent standard error of the mean. Abbreviations: DDT, dichlorodiphenyltrichloroethane; NM, no mortality.

The *An. gambiae* s.l. population exhibited high resistance to permethrin (type I pyrethroid; 0% mortality), DDT (organochlorine; 0%), the nonester pyrethroid etofenprox (2.6% ± 2.6%), and the type II pyrethroid λ-cyhalothrin (20.9% ± 3.5%), but moderate resistance to the type II pyrethroid deltamethrin (67.2% ± 7.5%) (Figure 1A). Resistance was also observed against the organochlorine dieldrin (66.6% ± 8.5% mortality). Full susceptibility was obtained with the organophosphates malathion and pirimiphos-methyl. *An. gambiae* s.l. females were fully susceptible to the carbamate propoxur, whereas males were slightly resistant (90.4% ± 1.7% mortality). Resistance of *An. gambiae* s.l. to bendiocarb, an insecticide commonly used in indoor residual spraying, was also assessed showing a low mortality level after a 30-minute (9.3% ± 4.8%) and 60-minute (78.7% ± 9.2%) exposure, but full susceptibility at a 90-minute exposure. Due to the high resistance observed with permethrin and DDT, bioassays with exposure times of 3 and 6 hours were performed (Figure 1B). Surprisingly, the mortality barely increased for DDT after 6 hours of exposure (3 hours: 2.8% ± 1.4%; 6 hours: 4.2% ± 2.1%), whereas it increased moderately for permethrin (3 hours: 21.8% ± 4.0%; 6 hours: 71.8% ± 7.8%).

**Figure 2.**
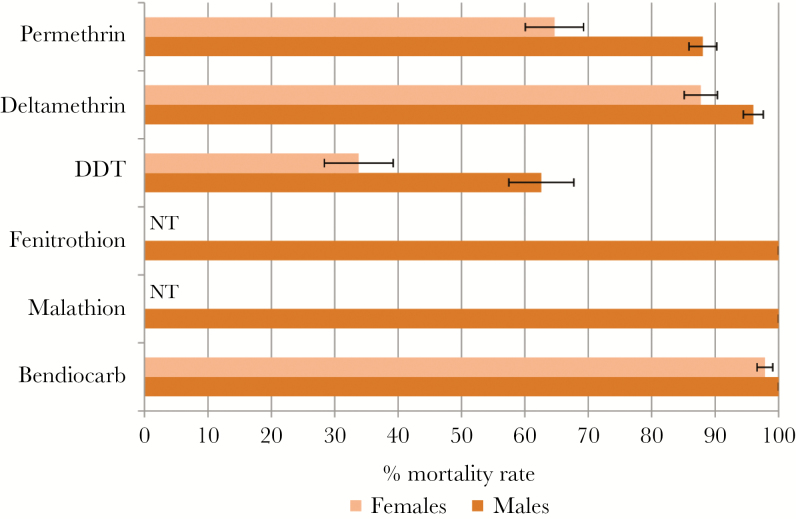
Susceptibility profile of *Anopheles funestus* sensu stricto population using World Health Organization insecticide susceptibility tube assays in Kinshasa. Error bars represent standard error of the mean. Abbreviations: DDT, dichlorodiphenyltrichloroethane; NT, not tested.

*An. funestus* s.s. was resistant to DDT (33.8% ± 5.4% mortality) and to permethrin (64.7% ± 4.6%), moderately resistant to deltamethrin (87.7% ± 2.6%), and nearly susceptible to bendiocarb (97.9% ± 1.2%). Only males were tested for fenitrothion and malathion, with a full susceptibility observed (Figure 2).

### Synergist Assays

**Figure 3.**
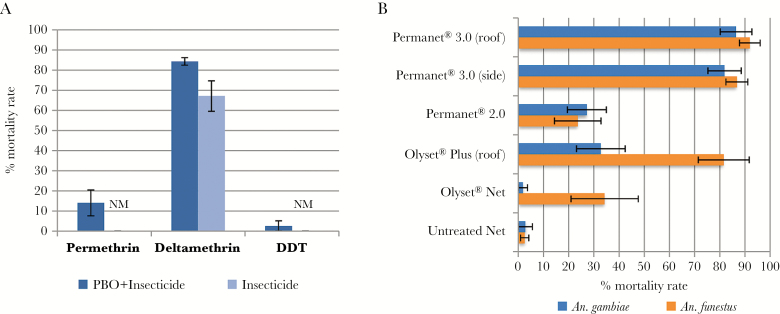
Synergist and bed net efficacy tests. *A*, Susceptibility profile of *Anopheles gambiae* sensu lato after synergist assay with piperonyl butoxide. *B*, Bioefficacy of different commercial long-lasting insecticidal nets against *An. gambiae* sensu lato and *Anopheles funestus* sensu stricto. Error bars represent standard error of the mean. Abbreviations: DDT, dichlorodiphenyltrichloroethane; NM, no mortality; PBO, piperonyl butoxide.

The synergist assay results showed a slight recovery of susceptibility after PBO preexposure for the 2 pyrethroids tested (permethrin: no PBO preexposure [0 ± 0%] mortality vs PBO preexposure [14.0% ± 6.5%], *P* = .048; deltamethrin: no PBO preexposure [67.1% ± 7.5%] vs PBO preexposure [84.4% ± 1.9%], *P* = .12), suggesting that cytochrome P450 enzymes may play a minor role in pyrethroid resistance in this population of *An. gambiae* s.l. (Figure 3A). Tests with DDT also revealed a lack of impact of PBO preexposure with only 2.7% ± 2.7% mortality (*P* = .29) observed with PBO exposure vs no mortality without exposure, suggesting that DDT resistance is conferred by other mechanisms or gene families. No mortality was observed in control mosquitoes exposed to the synergist PBO only.

### Insecticide-Treated Bed Net Efficacy

Cone assays were conducted to evaluate the efficacy of conventional bed nets (Figure 3B). A low efficacy of both OlysetNet and PermaNet 2.0 was observed for both mosquito species (*An. gambiae* s.l.: OlysetNet: 1.8% ± 1.8% mortality, PermaNet 2.0: 27.2% ± 7.2%; *An. funestus* s.s.: OlysetNet: 34.3% ± 13.4%, PermaNet 2.0: 23.6% ± 9.3%). The efficacy of the bed nets treated with PBO, OlysetPlus, and PermaNet 3.0 increased markedly for *An. funestus* s.s. (OlysetPlus: 81.6% ± 10.1% mortality, PermaNet 3.0-side: 86.8% ± 4.3%, PermaNet 3.0-roof: 91.9% ± 4.1%). For *An. gambiae* s.l., a higher efficacy was observed for both panels of the PermaNet 3.0 (PermaNet 3.0-side: 81.9% ± 6.6% mortality, PermaNet 3.0-roof: 86.5% ± 6.3%) than with the OlysetPlus (32.8% ± 9.6%). This difference is in line with the resistance profile showing a higher resistance level for permethrin, used for OlysetPlus impregnation, than for deltamethrin, used by PermaNet 3.0. Furthermore, for both species, OlysetPlus and PermaNet 3.0 are considerably more effective than OlysetNet and PermaNet 2.0 in cone bioassays, when unwashed.

### Polymorphism Analysis of the VGSC Gene in *An. gambiae*

**Figure 4.**
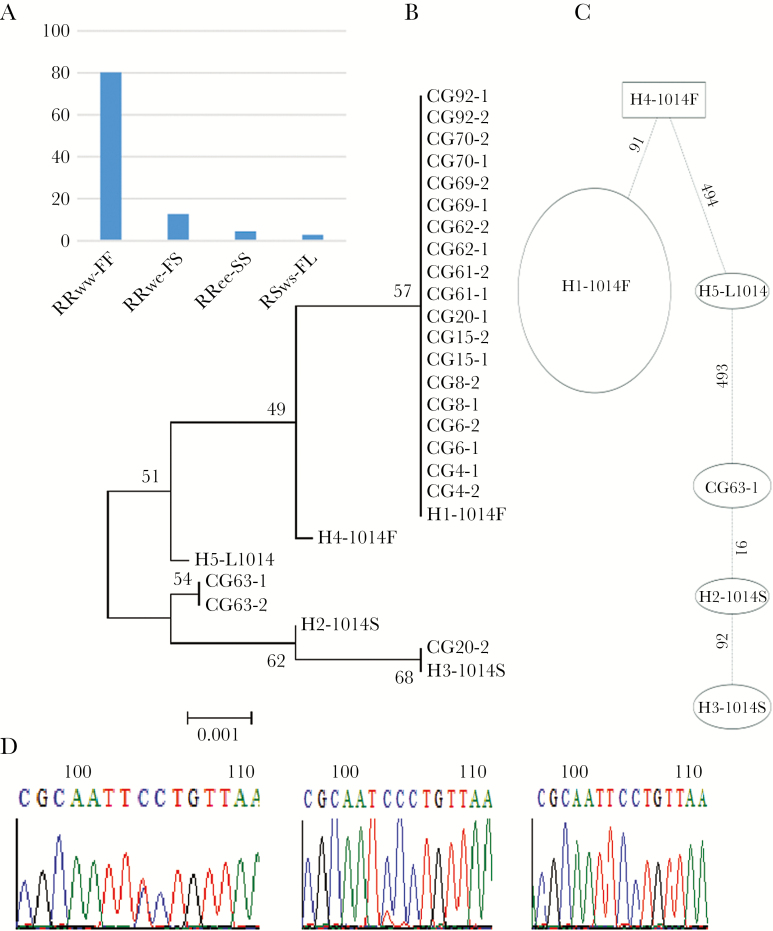
Analysis of the polymorphism of a portion of the voltage-gated sodium channel (VGSC) gene spanning the L1014F/S mutation. *A*, Distribution of the genotypes at the 1014 codon position. *B*, Maximum likelihood phylogenetic tree of VGSC fragment with previously recorded 1014F/S haplotypes across Africa [21]. *C*, Templeton-Crandall-Singh network for the VGSC haplotypes between susceptible and resistant permethrin samples in Kinshasa. Haplotypes are represented as an oval or a rectangle, scaled to reflect their frequencies. Lines connecting haplotypes and each node represent a single mutation event (respective polymorphic positions are given above branches). *D*, Sequencing traces showing the polymorphic positions 91 and 92 generating the third 1014S haplotype newly detected in Congo and suggesting an independent occurrence of 1014S.

The TaqMan genotyping of *kdr* target-site resistance mutations in VGSC, which confers resistance to pyrethroids and DDT in *An. gambiae* s.l. [27, 28], revealed that the 1014F *kdr* allele was at a high frequency (87.8% [195/222]), the 1014S allele frequency was lower (10.8% [24/222]), and the susceptible allele was virtually absent (1.4% [3/222]) (Figure 4A).

In addition, sequencing of a 510-bp fragment of VGSC gene spanning the 1014 codon revealed a reduced genetic diversity with only 4 polymorphic sites and 3 haplotypes, including a predominant 1014F haplotype (19/22), in line with the near fixation of the 1014F allele in this population. Comparison of the DRC-VGSC haplotypes with 4 *kdr* bearing haplotypes previously detected across Africa revealed that all 1014F haplotypes from Kinshasa belong to the H1-1014F haplotype, predominant in West/Central Africa [21] (Figure 4B). The 1014S haplotypes belong to the H3-1014S haplotype previously described in East Africa [21], whereas the other haplotype (CG63) exhibited a single mutational step difference (position 91) from the previously described H2-1014S. This is further supported by the Templeton-Crandall-Singh haplotype network tree (Figure 4C), showing that the 1014S haplotypes are separated by 1 or 2 confirmed mutational steps (Figure 4D), suggesting an independent occurrence of the CG63-1014S haplotype in DRC, potentially from local selection.

### Genotyping *An. funestus* L119F-GSTe2 and A296S-RDL Resistant Markers

**Figure 5.**
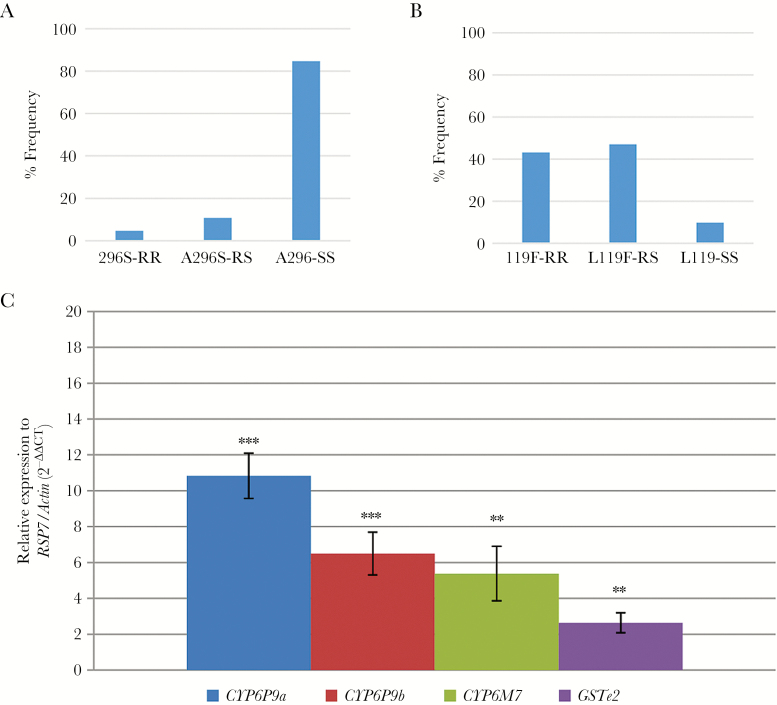
Investigation of molecular basis of resistance in *Anopheles funestus*. *A*, Distribution of the genotypes at the A296S RDL resistance marker. *B*, Distribution of the genotypes at the L119F resistance marker of the GSTE2 gene. *C*, Differential expression by quantitative reverse-transcription polymerase chain reaction of the major insecticide resistance genes in *An. funestus* sensu stricto in Kinshasa compared with the susceptible *An. funestus* sensu stricto strain FANG. Error bars represent standard error of the mean. **P < 0.01; ***P < 0. 001.

In *An. funestus* s.s., the A296S-RDL GABA receptor mutation which is known to confer resistance to dieldrin [29], was detected. However, the 296S resistant (R) allele frequency was low (10%) (Figure 5A). In contrast, for the L119F-GSTe2 marker conferring metabolic resistance to DDT/permethrin [23], a high frequency of the resistant 119F-GSTe2 (R) allele was observed (66.7%); 43.1% of the individuals genotyped were RR and 47.1% were RS (Figure 5B).

### Transcription Profile of Resistance Genes in *An. funestus*

The transcription analyses of 3 cytochrome P450 genes, *CYP6P9a*, *CYP6P9b*, and *CYP6M7*, known to be involved in pyrethroid resistance in *An. funestus* [30–32], and 1 glutathione-s-transferase, *GSTe2*, previously shown to confer DDT and permethrin resistance [23], were assessed by qRT-PCR in nonexposed mosquitoes (Figure 5C). The results reveal that these genes are significantly up-regulated in the field-collected *An. funestus* s.s., in comparison with the susceptible laboratory strain FANG.

## DISCUSSION

### Contribution of Vectors to Malaria Transmission

The high number of *An. gambiae* and *An. funestus* infected with *Plasmodium* (37.6% and 30.9%, respectively) suggests a high burden of malaria in this location. These infection rates are far higher than those obtained by Coene in 1993 in urban and rural areas of Kinshasa using the enzyme-linked immunosorbent assay (ELISA) detection method [33]. However, it should be noted that we were detecting all stages of the development of *Plasmodium*, whereas ELISA detects only the infective sporozoite stage. Furthermore, the infection rates observed in Kinshasa are considerably higher than those recently reported in other locations using the same method (Benin [18.2%], Ghana [12.5%], Malawi [5.3%], Nigeria [8.0%], and Uganda [5.3%]), as well as those obtained using other approaches (Guinea-Bissau [12.4%], Kenya [10%–18.5%], and Cameroon [6.5%–8.1%]) [13, 24, 34–39]. Our findings suggest that *P. falciparum* is the predominant malaria parasite in Kinshasa, although control and elimination efforts should not ignore *P. malariae* and *P. ovale*, present at low frequency, as previously reported [1, 40].

### Multiple Insecticide Resistance in Malaria Vector in Kinshasa

This study revealed a high frequency of resistance to multiple insecticide classes in *An. gambiae* and *An. funestus* which, together with their high level of *Plasmodium* infection rate, calls for urgent actions to be taken to control malaria in this region. Both malaria vectors exhibit resistance toward pyrethroids, the only insecticide class recommended for use on LLINs [41]. The insecticide resistance pattern observed in *An. gambiae* differs from that found in the locality of Kingasani (province of Kinshasa) in 2009, which showed higher mortalities [1, 3]. This difference in resistance may be due to the effect of environmental and physiological factors such as the temporal selection caused by insecticide pressures, as well as to the genetic heterogeneity of the mosquito populations in DRC. The Kinshasa *An. funestus* population also exhibits resistance to pyrethroids and DDT, but at a lower level than *An. gambiae.* In contrast, *An. funestus* is susceptible to the carbamate bendiocarb. The resistance profile of this *An. funestus* population is similar to the situation observed in East Africa, where full susceptibility to bendiocarb is reported [7, 18]. As observed in other African populations of malaria vectors, organophosphates are the only insecticide class to which there is full susceptibility, and these could be recommended for indoor residual spraying around Kinshasa.

The commonly used OlysetNet and Permanet 2.0 LLINs presented a very low bioefficacy under laboratory conditions. The low efficacy of OlysetNet, treated with permethrin only, is particularly evident against *An. gambiae*. This observation correlates well with the very high permethrin resistance observed for *An. gambiae* in Kinshasa. A previous assessment of efficacy of the OlysetNet in other parts of Kinshasa had reported a higher efficacy of the OlysetNet, suggesting either a heterogeneity of the response to this net or that resistance has worsened with time [5]. Even when the synergist PBO is combined with permethrin (OlysetPlus), the mortality of *An. gambiae* was low, indicating that P450 genes might not be the major drivers of the observed permethrin resistance, but rather the *kdr* mutation, which is nearly fixed in this population.

### Molecular Mechanisms Involved in Insecticide Resistance

The extremely high resistance to permethrin and DDT in *An. gambiae* correlates with the high frequency of the L1014F *kdr* mutation (87.8%). This mutation has been previously detected in DRC [3, 5]. Furthermore, the fact that 17% of mosquitoes possess the 1014S resistance allele further contributes to maintain the high resistance level. The likely independent selection of the 1014S mutation suggests that both migration and local selection forces are driving the development of pyrethroid/DDT resistance in DRC. A de novo occurrence of *kdr* mutations has previously been described across Africa with two 1014F and two 1014S haplotypes reported [21]. Similar to the low efficacy of Olyset Plus, the lack of notable recovery after PBO exposure in the synergist assays further supports the assumption that cytochrome P450s only play a minor role in this resistance, with *kdr* mutations likely driving resistance to pyrethroids/DDT. Although both type I and II pyrethroids have the same molecular target, the VGSC, resistance to permethrin (type I) was higher than to deltamethrin (type II). Several studies based on modeling, electrophysiology, and in vivo bioassay analyses have shown that the different L1014 polymorphisms may present different responses to the pyrethroids type I and type II [42–44]. For *An. gambiae*, similar results have been reported in a population with a high 1014F frequency in Burkina Faso (West Africa) [45], but also in Tanzania and Uganda (East Africa), where the presence of L1014S is predominant [46, 47]. Nevertheless, it is necessary to bear in mind that the differences in response to the type of pyrethroids may be also influenced by other minor mutations in the VGSC that may enhance the level of resistance associated with L1014 polymorphism [43, 48].

In the absence of *kdr* mutations in *An. funestus* s.s. [49], this study explored the contribution of metabolic resistance in the multiple resistance observed. Previous reports have confirmed the important role of the duplicated *CYP6P9a*/*CYP6P9b* and *CYP6M7* genes in pyrethroid resistance [30, 31]. The significant up-regulation of these genes in this population supports the predominant role of metabolic enzymes in the pyrethroid resistance. Nevertheless, the FC values obtained here are considerably lower than in southern African populations [24], suggesting that mechanisms are different as recently shown between African regions for this species [50]. On the other hand, up-regulation for *GSTe2* gene and the action of *L119F-GSTe2* mutation have been associated mainly with DDT resistance [23]. The *119F-GSTe2* mutation, which has been shown to play a key role in DDT resistance in West and Central Africa (23), was found in high frequency, which is consistent with the DDT resistance observed in Kinshasa. Due to this, and along with the high frequency of *kdr* in *An. gambiae*, the use of DDT as an alternative to pyrethroids would not be recommended for vector control. Additionally, the presence of the 296S-RDL resistant to dieldrin allele is reported for the first time in DRC and could be a consequence of past use of this insecticide or ongoing use in the agricultural sector.

## CONCLUSIONS

Besides the high *Plasmodium* infection rate in both *An. funestus* and *An. gambiae*, the results from this study reveal high and multiple insecticide resistance patterns, together with an alarmingly low efficacy of conventional LLINs without the synergist PBO under laboratory conditions. This study highlights the urgent need for actions to better manage the issue of insecticide resistance in order to prolong the effectiveness of the ongoing and future malaria control programs in DRC.

## Notes

***Author contributions.*** C. S. W. designed the study. J. M. R., H. I., and C. S. W. performed the experiments and analyzed the data. C. S. W., S. I., and F. T. W. supervised the fieldwork. J. M. R., S. I., and C. S. W. wrote the manuscript.

***Acknowledgments.*** John Gimnig is thanked for his critical reading of the manuscript.

***Financial support.*** This work was supported by a Wellcome Trust Senior Research Fellowship in Biomedical Sciences to C. S. W. (award number 101893/Z/13/Z). S. I. is funded by the US President’s Malaria Initiative.

***Potential conflicts of interest.*** All authors: No reported conflicts of interest. All authors have submitted the ICMJE Form for Disclosure of Potential Conflicts of Interest. Conflicts that the editors consider relevant to the content of the manuscript have been disclosed.
